# Integration of Mouse and Human Genome-Wide Association Data Identifies *KCNIP4* as an Asthma Gene

**DOI:** 10.1371/journal.pone.0056179

**Published:** 2013-02-14

**Authors:** Blanca E. Himes, Keith Sheppard, Annerose Berndt, Adriana S. Leme, Rachel A. Myers, Christopher R. Gignoux, Albert M. Levin, W. James Gauderman, James J. Yang, Rasika A. Mathias, Isabelle Romieu, Dara G. Torgerson, Lindsey A. Roth, Scott Huntsman, Celeste Eng, Barbara Klanderman, John Ziniti, Jody Senter-Sylvia, Stanley J. Szefler, Robert F. Lemanske, Robert S. Zeiger, Robert C. Strunk, Fernando D. Martinez, Homer Boushey, Vernon M. Chinchilli, Elliot Israel, David Mauger, Gerard H. Koppelman, Dirkje S. Postma, Maartje A. E. Nieuwenhuis, Judith M. Vonk, John J. Lima, Charles G. Irvin, Stephen P. Peters, Michiaki Kubo, Mayumi Tamari, Yusuke Nakamura, Augusto A. Litonjua, Kelan G. Tantisira, Benjamin A. Raby, Eugene R. Bleecker, Deborah A. Meyers, Stephanie J. London, Kathleen C. Barnes, Frank D. Gilliland, L. Keoki Williams, Esteban G. Burchard, Dan L. Nicolae, Carole Ober, Dawn L. DeMeo, Edwin K. Silverman, Beverly Paigen, Gary Churchill, Steve D. Shapiro, Scott T. Weiss

**Affiliations:** 1 Channing Division of Network Medicine, Brigham and Women’s Hospital and Harvard Medical School, Boston, Massachusetts, United States of America; 2 Children’s Hospital Informatics Program, Boston, Massachusetts, United States of America; 3 Partners HealthCare Center for Personalized Genetic Medicine, Boston, Massachusetts, United States of America; 4 The Jackson Laboratory, Bar Harbor, Maine, United States of America; 5 Department of Medicine, University of Pittsburgh School of Medicine, Pittsburgh, Pennsylvania, United States of America; 6 Department of Human Genetics, University of Chicago, Chicago, Illinois, United States of America; 7 Department of Medicine, University of California San Francisco, San Francisco, California, United States of America; 8 Department of Public Health Sciences, Henry Ford Health System, Detroit, Michigan, United States of America; 9 Department of Preventative Medicine, University of Southern California, Los Angeles, California, United States of America; 10 Department of Medicine, Johns Hopkins University, Baltimore, Maryland, United States of America; 11 International Agency for Research on Cancer (IARC), Lyon, France; 12 National Jewish Health and University of Colorado Denver School of Medicine, Denver, Colorado, United States of America; 13 University of Wisconsin School of Medicine and Public Health, Madison, Wisconsin, United States of America; 14 Kaiser Permanente Southern California Region, San Diego, California, United States of America and University of California San Diego, La Jolla, California, United States of America; 15 Washington University School of Medicine, St. Louis, Missouri, United States of America; 16 Arizona Respiratory Center, University of Arizona, College of Medicine, Tucson, Arizona, United States of America; 17 Division of Pulmonary/Critical Care and Allergy/Immunology, Department of Medicine, University of California San Francisco, San Francisco, California, United States of America; 18 Department of Public Health Sciences, Penn State College of Medicine, Hershey, Pennsylvania, United States of America; 19 Division of Pulmonary and Critical Care Medicine, Department of Medicine, Brigham and Women’s Hospital, Boston, Massachusetts, United States of America; 20 Department of Pediatric Pulmonology and Pediatric Allergology, Beatrix Children’s Hospital, GRIAC Research Institute, University of Groningen, University Medical Center Groningen, Groningen, The Netherlands; 21 Department of Pulmonology and Tuberculosis, GRIAC Research Institute, University of Groningen, University Medical Center Groningen, Groningen, The Netherlands; 22 Department of Epidemiology, GRIAC Research Institute, University of Groningen, University Medical Center Groningen, Groningen, The Netherlands; 23 Nemours Children's Clinic, Center for Pharmacogenomics and Translational Research, Jacksonville, Florida, United States of America; 24 Vermont Lung Center, Department of Medicine and Physiology, University of Vermont, Burlington, Vermont, United States of America; 25 Center for Genomics and Personalized Medicine Research, Wake Forest School of Medicine, Winston-Salem, North Carolina, United States of America; 26 RIKEN Center for Genomic Medicine, Kanagawa, Japan; 27 Laboratory of Molecular Medicine, The Institute of Medical Science, The University of Tokyo, Tokyo, Japan; 28 National Institute of Environmental Health Sciences, National Institutes of Health, Department of Health and Human Services, Research Triangle Park, North Carolina, United States of America; 29 Center for Health Policy and Health Services Research, Department of Internal Medicine, Henry Ford Health System, Detroit, Michigan, United States of America; Yale University, United States of America

## Abstract

Asthma is a common chronic respiratory disease characterized by airway hyperresponsiveness (AHR). The genetics of asthma have been widely studied in mouse and human, and homologous genomic regions have been associated with mouse AHR and human asthma-related phenotypes. Our goal was to identify asthma-related genes by integrating AHR associations in mouse with human genome-wide association study (GWAS) data. We used Efficient Mixed Model Association (EMMA) analysis to conduct a GWAS of baseline AHR measures from males and females of 31 mouse strains. Genes near or containing SNPs with EMMA p-values <0.001 were selected for further study in human GWAS. The results of the previously reported EVE consortium asthma GWAS meta-analysis consisting of 12,958 diverse North American subjects from 9 study centers were used to select a subset of homologous genes with evidence of association with asthma in humans. Following validation attempts in three human asthma GWAS (i.e., Sepracor/LOCCS/LODO/Illumina, GABRIEL, DAG) and two human AHR GWAS (i.e., SHARP, DAG), the Kv channel interacting protein 4 (*KCNIP4*) gene was identified as nominally associated with both asthma and AHR at a gene- and SNP-level. In EVE, the smallest *KCNIP4* association was at rs6833065 (P-value 2.9e-04), while the strongest associations for Sepracor/LOCCS/LODO/Illumina, GABRIEL, DAG were 1.5e-03, 1.0e-03, 3.1e-03 at rs7664617, rs4697177, rs4696975, respectively. At a SNP level, the strongest association across all asthma GWAS was at rs4697177 (P-value 1.1e-04). The smallest P-values for association with AHR were 2.3e-03 at rs11947661 in SHARP and 2.1e-03 at rs402802 in DAG. Functional studies are required to validate the potential involvement of *KCNIP4* in modulating asthma susceptibility and/or AHR. Our results suggest that a useful approach to identify genes associated with human asthma is to leverage mouse AHR association data.

## Introduction

Asthma is a common chronic respiratory disease with a rise in prevalence over the past decades, affecting over 25 million Americans and 300 million people world-wide [1], [2]. Asthma is characterized by airway hyperresponsiveness (AHR), a trait distinguished by increased airway smooth muscle contractility in response to certain exposures. The mouse is commonly used to model the genetics of human diseases because mice are physiologically similar to humans, the mouse genome has been sequenced, and many tools exist that allow for direct testing of genetic alterations in mice [3], [4]. Although attempts to recreate all of the features of human asthma in mice have not been successful [5], many of the allergic asthmatic responses in mouse are similar to the responses observed in humans [6], [7]. The most common asthma phenotype studied in the mouse is AHR, and several regions that have been associated with mouse AHR are homologous with genomic regions linked with asthma-related phenotypes in human cohorts. For example, two linkage studies [8], [9] in mouse identified AHR quantitative trait loci (QTL) on chromosome 7 that are homologous with human AHR QTL on chromosome 19q observed in Hutterites and Chinese individuals [10], [11]. Mouse AHR QTL observed on chromosome 17 in three studies [9], [12], [13] overlap with human chromosome 6p QTLs identified in four studies of asthma and allergic phenotypes [14], [15], [16], [17]. Thus, it is likely that mouse and human share genetic variants that predispose both species to asthma-related phenotypes, and mouse AHR is an appropriate phenotype to identify some of these shared genetic variants.

Genome-wide association analysis of mouse inbred strains can provide a cost effective complement to traditional QTL methods [18], [19]. Such methods leverage the fact that laboratory mouse strains, whose lineage is well known, are nearly isogenic due to their inbred origin [20] and therefore, share large haplotype blocks that are identical by descent. Assuming that phenotypic differences observed between mouse strains are due to different variants inherited within ancestral haplotypes, regions associated with a trait can be narrowed by comparison of haplotypes across many strains. The ability to narrow such regions efficiently is made possible by genetic resources and bioinformatics tools, including dense SNP sets for mouse strains [21], [22] and association algorithms [18], [19]. Studies using mouse strain surveys have been successful in identifying genes relevant to human disease, such as HDL levels in blood [18]. A previous *in silico* QTL study of baseline AHR in 36 female mouse strains identified eight possible regions that could underlie differences in AHR among strains [23]. The results of this study have not been extensively validated or evaluated in humans.

The genetic basis of asthma has been widely studied in humans [24]. Most recently, multi-center, multi-cohort genome-wide association studies (GWAS) of asthma have been carried out in Europeans and diverse North American populations [25], [26]. These and other GWAS have begun to identify loci (e.g. *IKZF3-ZPBP2-GSDMB-ORMDL3* locus, *HLA-DQ, IL1RL1, IL18RL1, IL33, TSLP, SLC22A5, SMAD3,* and *RORA*) that are consistently associated with asthma at statistical thresholds that leave little doubt the results are truly significant. However, it is likely that some of the findings that do not meet genome-wide significance levels in these GWAS represent true associations that are biologically relevant for asthma. The question of how to distinguish true associations among the false positive ones is challenging. In this work, we address this challenge by using mouse baseline AHR association measures and searching among previously published nominally significant EVE Consortium GWAS meta-analysis results [26] to identify genes that may be associated with asthma. After attempting to confirm our results in three additional asthma GWAS and two AHR GWAS, we found that the Kv channel interacting protein 4 (*KCNIP4*) is likely to be related to asthma and AHR.

## Results

**Figure 1:**
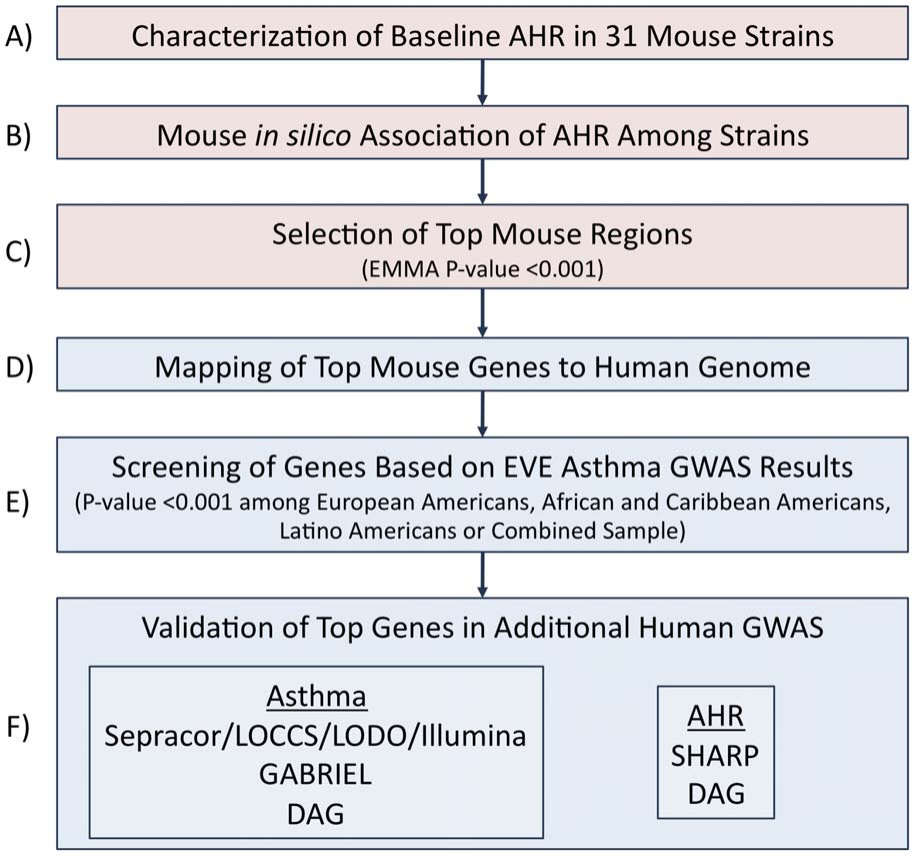
Study overview. Mouse association study (in pink): (A) Baseline AHR slope measures were obtained for 31 mouse strains, and these were (B) used to perform an association study using EMMA software. (C) Mouse genes near or containing SNPs with p-values <0.001 were selected for follow-up study in human GWAS datasets. Integration of mouse results with human data (in blue): (D) HomoloGene maps were used to obtain human homologous genes corresponding to the top mouse genes. (E) Genes with SNPs having p-values <0.001 in the combined EVE meta-analysis or within race/ethnic specific GWAS were selected for (F) replication in other human GWAS.

**Figure 2:**
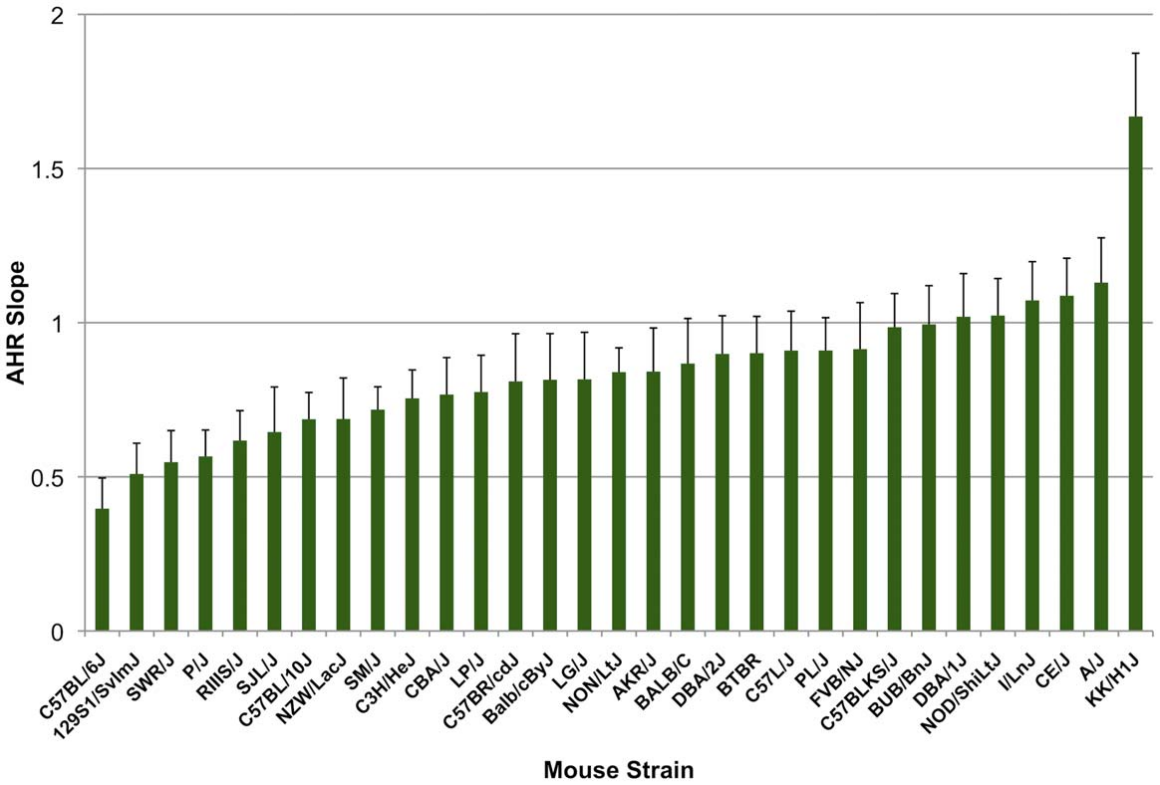
Baseline AHR slope phenotype distribution among 31 mouse strains. The AHR slope, displayed along the y-axis, was computed as the slope of the resistance measures vs. log-transformed methacholine concentrations for each mouse strain. Names for each mouse strain are displayed along the x-axis.

**Figure 3:**
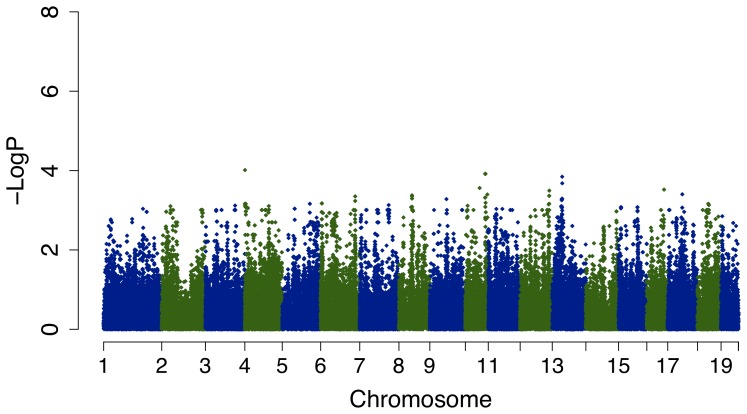
Manhattan plot of mouse AHR slope EMMA results. The x-axis denotes position along each chromosome. The y-axis denotes –Log_10_(P) corresponding to EMMA P-values. None of the P-values are significant after corrections for multiple comparisons, but all those with nominal P-values <0.001 were selected for further study.

Figure 1 is an overview of our study design. Measures of baseline AHR in 31 mouse strains found that there was up to a 4.2-fold change in AHR slope across strains, with the smallest slope (i.e. 0.40 SEM 0.10) corresponding to the C57BL/6J strain and the largest slope (i.e. 1.67 SEM 0.20) corresponding to the KK/H1J strain [Figure 2]. The complete AHR phenotype results are freely available as project “Berndt1” in the Mouse Phenome Database of the Jackson Laboratory. The EMMA results for AHR slope did not contain any P-values that were significant after correction for multiple comparisons (i.e. <0.05/281,300 = 1.8e-07) [Figure 3]. Nonetheless, some genomic regions had P-values that were nominally significant and may indicate true associations with AHR slopes. The set of 227 mouse genes within 50 kb of SNPs with EMMA P-values <0.001 was selected for further study.

**Figure 4:**
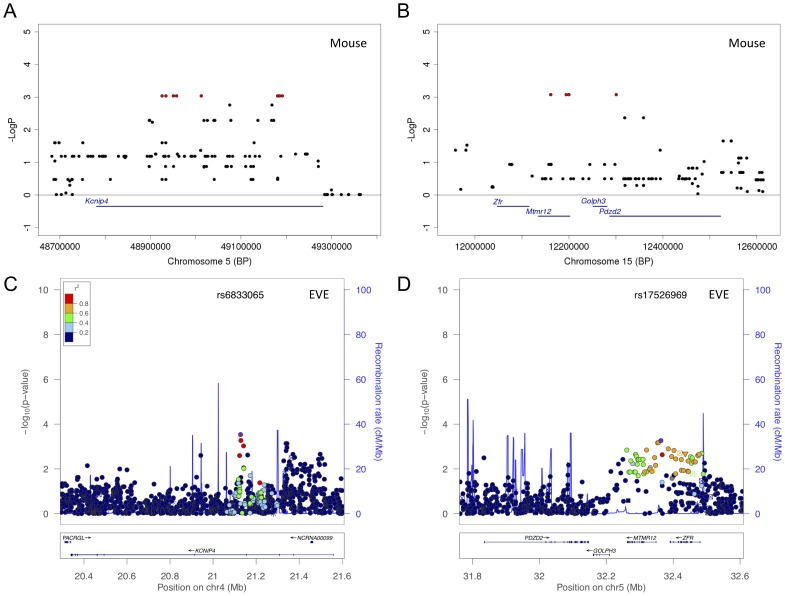
Top regions of association in mouse and human. Mouse plots near (A) *Kcnip4* and (B) *Pdzd2/Golph3/Mtmr12/Zfr* contain –Log_10_ of EMMA p-values vs. position along the corresponding mouse chromosome. Corresponding human homologous plots near (C) *KCNIP4* and (D) *PDZD2/GOLPH3/MTMR12/ZFR*. The x-axis denotes position along corresponding human chromosome, while the y-axis denotes –Log_10_(P) corresponding to EVE p-values for the combined sample GWAS (C) or European American GWAS (D). LD between the SNPs with the lowest P-value to other SNPs in the human plots are denoted in colors and were computed according to HapMap Phase 2 CEU data using LocusZoom [55].

**Figure 5:**
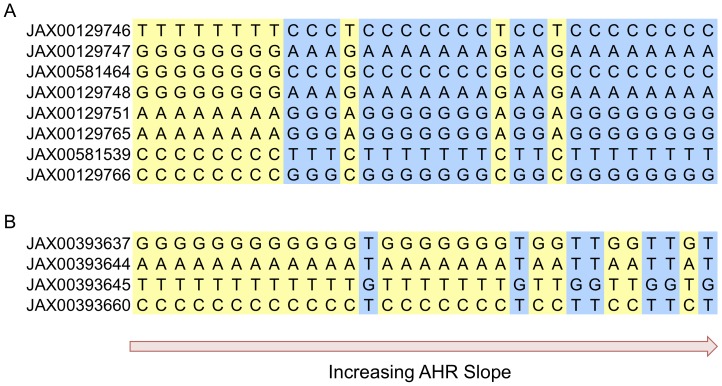
Mouse genotypes for SNPs with P-value <0.001 in the top two association regions. A) *Kcnip4* and B) *Pdzd2/Golph3/Mtmr12/Zfr.* Each column corresponds to a mouse strain, ordered as displayed in Figure 2, such that AHR slope increases from left to right.

Of the 227 top mouse genes, 145 had human orthologs, according to the human-mouse HomoloGene map utilized. Results from the EVE GWAS based on all samples and specific to race/ethnic groups were obtained for all SNPs within 50 kb of the set of 145 genes. A subset of 35 genes had at least one SNP with a P-value <0.001 in the EVE European American, African and Caribbean American, Latino American and/or all subjects’ GWAS. The results in the vicinity of these 35 genes were explored more carefully to ensure that 1) the AHR slope phenotype distribution approximately matched the genotype distribution across mouse strains, 2) the mouse association results were based on SNPs with minor allele frequency >2/31, and 3) the association results were supported by a region of LD in both mouse and human. Two regions that included a total of five genes met these criteria: 1) *KCNIP4*, a 1220.19 kb-long gene that was covered (including 50 kb on either end) by 1,766 EVE combined meta-analysis SNPs, and 2) *PDZD2/GOLPH3/MTMR12/ZFR*, a 732 kb-long region that was covered (including 50 kb on either end) by 697 EVE European American analysis SNPs [Figure 4]. The smallest mouse EMMA p-values for these two regions were 9.1e-04 for *Kcnip4* and 8.4e-04 for *Pdzd2/Golph3/Mtmr12/Zfr*. The genotype distribution for mouse SNPs with these P-values in the corresponding regions are shown in Figure 5. The EVE P-values supporting the *KCNIP4* association were from the combined meta-analysis, and the lowest P-value (2.9e-04) was for SNP rs6833065. The EVE P-values supporting the *PDZD2/GOLPH3/MTMR12/ZFR* region of association were from the European American results, and the lowest P-value (5.5e-04) was for SNP rs17526969.

**Figure 6:**
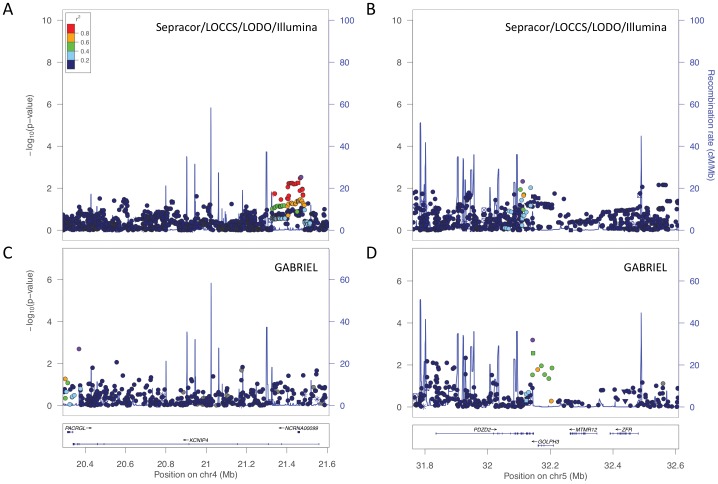
Top regions of association in two independent human asthma GWAS. *KCNIP4* association in A) Sepracor/LOCCS/LODO/Illumina and C) GABRIEL. *PDZD2/GOLPH3/MTMR12/ZFR* association in B) Sepracor/LOCCS/LODO/Illumina and D) GABRIEL. The x-axis denotes position along corresponding human chromosome, while the y-axis denotes –Log_10_(P). LD between the SNPs with the lowest P-value to other SNPs in the plots are denoted in colors and were computed according to HapMap Phase 2 CEU data using LocusZoom [55].

We next attempted to replicate the asthma association findings in two human cohorts: Sepracor/LOCCS/LODO/Illumina and GABRIEL. We found that there was gene level replication for *KCNIP4*, and, considering the genomic interval spanning four genes, some regional replication for the *PDZD2/GOLPH3/MTMR12/ZFR* association [Figure 6]. Each asthma GWAS had a different top hit in each region, and only *KCNIP4* showed evidence of SNP-level replication at a nominally significant threshold [Table 1]. Based on the replication results in GABRIEL and Sepracor/LOCCS/LODO/Illumina, we attempted to replicate the *KCNIP4* findings in DAG. The lowest EVE P-value of 2.9e-04 at rs6833065 did not replicate in Sepracor/LOCCS/LODO/Illumina or DAG, and data for this SNP was not available in GABRIEL. The lowest GABRIEL association at rs469177 (P-value 1.0e-03) was nominally significant in EVE and DAG, but not Sepracor/LOCCS/LODO/Illumina. The lowest Sepracor/LOCCS/LODO/Illumina association at rs7664617 (P-value 1.5e-03) was nominally significant in EVE, but data for this SNP was not available in GABRIEL or DAG. The lowest combined P-value across the four asthma GWAS was 1.1e-04 for rs4697177, while the three SNPs with nominally significant P-values in 3/4 GWAS were rs4697177, rs6448072, and rs6856781. The allelic frequencies and odds ratios for the top *KCNIP4* SNPs are shown in Table 2 and Table 3, respectively.

**Table 1:** Top *KCNIP4* SNP Associations.

SNP	CHR	BP	A1	A2	EVE P-value	GABRIEL P-value	SLLI P-value	DAG P-value	Combined Asthma P-value	SHARP AHR P-value	DAG AHR P-value
rs4697177	4	20369187	G	T	3.0E−02	**1.0E−03**	1.9E–01	2.3E–02	**1.1E–04**	6.7E–01	2.0E–01
rs4696975	4	20927269	A	T	1.5E–01	–	2.0E–01	**3.1E**–**03**	4.9E–03	3.3E–01	3.3E–01
rs6833065	4	21127809	C	T	**2.9E**–**04**	–	2.2E–01	2.2E–01	1.1E–03	9.1E–01	5.2E–01
rs2279674	4	21129348	G	T	5.4E–04	4.1E–01	2.3E–01	2.2E–01	3.6E–03	9.0E–01	5.2E–01
rs1870626	4	21141706	C	G	9.3E–04	–	2.2E–01	2.0E–01	2.5E–03	8.9E–01	5.1E–01
rs11947661	4	21163270	A	C	1.3E–01	–	4.6E–01	5.8E–01	3.4E–01	**2.3E**–**03**	1.4E–01
rs402802	4	21181943	A	G	4.1E–01	1.2E–01	7.8E–01	4.4E–01	4.1E–01	2.0E–01	**2.1E**–**03**
rs10034603	4	21339051	G	T	7.3E–04	–	4.3E–02	–	3.6E–04	7.0E–01	–
rs7378252	4	21343887	A	C	7.4E–04	–	3.9E–02	5.3E–01	1.1E–03	7.3E–01	6.9E–01
rs6448072	4	21352329	C	T	6.8E–03	4.6E–02	3.6E–02	5.3E–01	2.2E–03	8.3E–01	7.1E–01
rs6856781	4	21368864	C	T	1.1E–02	4.1E–02	3.8E–02	–	1.2E–03	7.2E–01	–
rs7664617	4	21471526	C	G	2.0E–02	–	**1.5E**–**03**	3.8E–01	8.9E–04	7.3E–01	4.1E–01

Table includes SNPs that have EVE P-value <0.001, are the lowest in individual GWAS (indicated in bold), or have nominally significant (i.e. P-value<0.05) replications in three of four asthma GWAS. P-values in asthma replication GWAS are 1-sided, based on the effect direction in EVE, to ensure that associations reported have effects in consistent directions. Fisher combined P-values are based on the populations with available results. *SLLI = Sepracor/LOCCS/LODO/Illumina.

**Table 2:** Allele Frequencies of Top *KCNIP4* SNP Associations.

SNP	Reference Allele	EVE EA Cases	EVE EA Controls	EVE AA Cases	EVE AA Controls	EVE LA Cases	EVE LA Controls	GABRIEL	SLLI*	DAG	SHARP AHR	DAG AHR
rs4697177	G	0.68	0.69	0.80	0.80	0.68	0.71	[0.62–0.74]	0.68	0.69	0.68	0.69
rs4696975	A	0.91	0.91	0.90	0.88	0.89	0.88	–	0.91	0.93	0.91	0.94
rs6833065	C	0.31	0.28	0.31	0.30	0.27	0.25	–	0.31	0.33	0.31	0.34
rs2279674	G	0.69	0.72	0.69	0.70	0.73	0.75	[0.63–0.75]	0.69	0.67	0.69	0.66
rs1870626	C	0.31	0.28	0.34	0.34	0.26	0.25	–	0.31	0.33	0.31	0.34
rs11947661	A	0.50	0.50	0.56	0.56	0.66	0.67	–	0.51	0.50	0.52	0.50
rs402802	A	0.47	0.45	0.42	0.43	0.57	0.56	[0.28–0.54]	0.47	0.42	0.44	0.42
rs10034603	G	0.62	0.64	0.35	0.37	0.39	0.37	–	0.64	–	0.62	–
rs7378252	A	0.38	0.36	0.74	0.72	0.61	0.63	–	0.36	0.36	0.38	0.36
rs6448072	C	0.62	0.64	0.74	0.78	0.41	0.40	[0.53–0.68]	0.64	0.64	0.63	0.64
rs6856781	C	0.38	0.36	0.19	0.16	0.58	0.60	[0.32–0.48]	0.36	–	0.38	–
rs7664617	C	0.48	0.51	0.15	0.15	0.32	0.30	–	0.51	0.49	0.47	0.48

Allele frequencies among EVE European Americans (EA), African and Caribbean Americans (AA), and Latino Americans (LA) are reported separately. Minimum and Maximum allele frequencies among GABRIEL populations are reported. For DAG, the mean allele frequency for subjects genotyped on two platforms is reported. For SLLI and SHARP, the frequencies among all subjects are reported. *SLLI = Sepracor/LOCCS/LODO/Illumina.

**Table 3:** Effect Sizes of Top *KCNIP4* SNP Associations.

SNP	Reference Allele	EVE	GABRIEL	SLLI	DAG	SHARP AHR	DAG AHR
rs4697177	G	0.95 [0.87, 1.03]	0.94 [0.90, 0.98]	0.92 (0.09)	0.87	0.98 [0.88, 1.09]	0.12
rs4696975	A	1.06 [0.93, 1.22]	–	1.13 (0.15)	1.44	1.10 [0.91, 1.32]	0.17
rs6833065	C	1.13 [1.03, 1.22]	–	1.07 (0.09)	1.06	1.01 [0.90, 1.13]	−0.06
rs2279674	G	0.88 [0.81, 0.96]	1.00 [0.95, 1.04]	0.93 (0.09)	0.95	0.99 [0.88, 1.11]	−0.06
rs1870626	C	1.12 [1.02, 1.21]	–	1.08 (0.09)	1.06	1.01 [0.90, 1.13]	−0.06
rs11947661	A	1.02 [0.94, 1.10]	–	1.01 (0.08)	0.99	0.84 [0.76, 0.94]	0.12
rs402802	A	1.04 [0.96, 1.13]	1.02 [0.98, 1.06]	0.94 (0.08)	1.01	1.07 [0.96, 1.19]	−0.27
rs10034603	G	0.92 [0.85, 1.00]	–	0.86 (0.09)	–	1.02 [0.92, 1.14]	–
rs7378252	A	1.07 [0.99, 1.16]	–	1.16 (0.09)	0.99	0.98 [0.88, 1.09]	−0.04
rs6448072	C	0.93 [0.85, 1.01]	0.97 [0.93, 1.01]	0.86 (0.09)	1.01	1.01 [0.91, 1.13]	−0.03
rs6856781	C	1.07 [0.98, 1.16]	1.04 [1.00, 1.08]	1.17 (0.09)	–	0.98 [0.88, 1.09]	–
rs7664617	C	0.94 [0.86, 1.03]	–	0.78 (0.09)	0.98	1.02 [0.92, 1.13]	0.07

Odds ratios are shown for asthma (EVE combined cohort, GABRIEL, Sepracor/LOCCS/LODO/Illumina (SLLI), and DAG) and for AHR (SHARP AHR for change in LnPC20, DAG AHR for change in Ln(Slope)). EVE, GABRIEL, and SHARP include 95% confidence intervals. SLLI includes standard errors.

**Figure 7:**
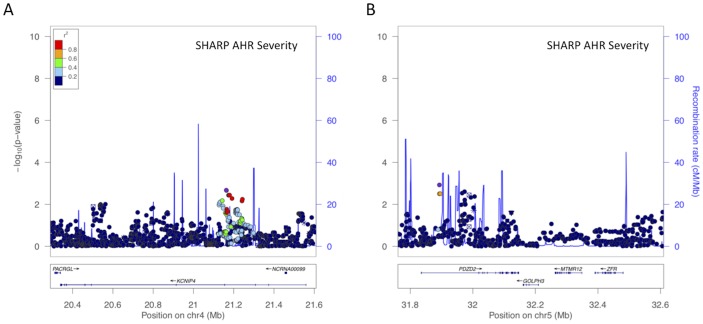
Top regions of association in a human AHR GWAS. (A) *KCNIP4* and (B) *PDZD2/GOLPH3/MTMR12/ZFR* associations in SHARP. The x-axis denotes position along corresponding human chromosome, while the y-axis denotes –Log_10_(P). LD between the SNPs with the lowest P-value to other SNPs in the plots are denoted in colors and were computed according to HapMap Phase 2 CEU data using LocusZoom [55].

Because the mouse phenotype measured, baseline AHR, is not a precise surrogate of human asthma, we also attempted to measure the association of top EMMA SNPs with another related phenotype for which GWAS data are available: human AHR among asthmatics. Of the top two regions, *KCNIP4* and *PDZD2/GOLPH3/MTMR12/ZFR,* only *KCNIP4* had gene-level replication in the SHARP AHR GWAS at a nominally significant level in the same regions as the asthma GWAS studies [Figure 7]. At a SNP level, there was no overlap between the strongest associations with asthma vs. AHR in either the SHARP or DAG AHR GWAS [Table 1].

## Discussion

Many risk-modifying loci have been identified via human GWAS studies for a wide range of complex diseases, including asthma [27]. Over time, the number of loci identified has grown, due in part to the greater statistical power of studies conducted. The primary way in which increased statistical power has been achieved is to conduct large-scale meta-analyses based on cohorts from many smaller studies. While the number of associations that pass genome-wide significance thresholds has increased, it is likely that some true associations that represent biologically important processes underlying diseases are present among the nominally significant ones. Approaches to identify true associations that are not among results meeting genome-wide significance levels in GWAS include attempts to replicate nominally significant findings in independent populations [26], [28], to conduct functional studies of top-ranked associations [29], [30], and to use gene-based [31] and pathway-based [32] methods. In this work, we have attempted to use a mouse genome-wide association analysis based on a strain survey of baseline AHR to identify asthma associations in humans.

The top two regions identified based on having mouse EMMA P-values <0.001 and EVE meta-analysis P-values <0.001 were *KCNIP4* and *PDZD2/GOLPH3/MTMR12/ZFR*. While there was some evidence of gene- or regional-level replication for both sites among the GABRIEL and Sepracor/LOCCS/LODO/Illumina asthma GWAS studies, only the *KCNIP4* gene had evidence of replication at individual SNPs. Additionally, only *KCNIP4* SNPs were nominally associated with AHR among asthmatics in the SHARP AHR GWAS, indicating that this gene may be related to both baseline AHR (i.e. AHR in the absence of asthma) and asthma. Thus, we selected *KCNIP4* as the final candidate for replication in DAG. We found that there was additional gene-level and SNP-level replication for *KCNIP4* with asthma in DAG, and gene-level replication for *KCNIP4* with AHR in DAG.

In addition to the nominal associations we observed, other studies have observed *KCNIP4* SNP associations with asthma-related phenotypes. First, a small GWAS of toluene diisocyanate-induced asthma in Koreans (84 cases and 263 controls) found that *KCNIP4* SNP rs4697192 had a P-value of 6.11e-05 for association under a recessive model [33]. Second, a family-based GWAS of change in lung function in response to glucocorticoid therapy among 118 asthmatics found that *KCNIP4* SNP rs4282162 had a P-value of 0.028 under a recessive model of inheritance, but attempts to replicate this result in three independent populations were unsuccessful [30]. Neither of these SNPs was among those considered in Table 1. In a follow-up study of the first EVE meta-analysis GWAS, all SNPs with meta-analysis P-values <0.001 in the combined sample or <1e-04 in the race/ethnic-specific analyses were selected for replication [28]. Five *KCNIP4* SNPs, including rs6833065 that is in Table 1, were among the SNPs genotyped in nine different replication populations of diverse origin (4 European American, 4 African American, 5 Latino American), comprised of 7202 cases, 6426 controls, and 507 case-parent trios. None of the *KCNIP4* SNPs replicated the primary EVE findings. Taken together, the *KCNIP4* association results from the current study and three previous ones suggest that if the nominally significant associations found represent a true relationship between this gene and asthma, then there is not a clear single functional variant underlying this relationship. Rather, there are weak effects observed at multiple sites that implicate the gene. It is possible that more thorough genotyping of *KCNIP4* in the populations where single or few SNPs did not replicate primary findings would uncover regions of association that further support gene- or SNP-level replication. Future studies may also be able to investigate whether *KCNIP4* variants are associated with AHR among non-asthmatics, as the mouse data found that *KCNIP4* variants were associated with baseline AHR.

Based on the mouse and human association results, the Kv channel interacting protein 4 (*KCNIP4*) emerged as the gene most likely to be related to asthma and AHR. Current information for this gene was gathered with the AceView tool [34]. *KCNIP4* is found on human chromosome 4, at 4p15.32, covering 1220.19 kb, from 21950417 to 20730227 (NCBI 37, August 2010). The protein encoded by *KCNIP4* was first identified in a study searching for proteins that interacted with presenilins, which are linked to early-onset Alzheimer’s disease [35]. *KCNIP4* was found to interact with the voltage-gated potassium channel subunit Kv4, as well as the presenilin PSEN2, and to be mostly expressed in brain. Based on its structure, *KCNIP4* belongs to the recoverin branch of calcium binding proteins, characterized by having 4 EF-hand motifs, the first of which does not bind calcium. In addition to being expressed in brain, *KCNIP4* is expressed in other tissues, including human skeletal muscle. Previous studies of peptides that bind to the EF-hands of calmodulin and other proteins that modulate intracellular calcium levels have found that these calcium sensors may be therapeutic targets for asthma [36]. For example, intratracheal pretreatment of guinea pigs with a calcium-like peptide (i.e. CALP1) that binds the EF-hands of calmodulin prior to ovalbumin challenge, prevented the development of AHR and attenuated the increased radical production by alveolar inflammatory cells of ovalbumin-challenged guinea pigs [37]. Thus, one possible mechanism by which *KCNIP4* may influence asthma and AHR is via modulation of intracellular calcium.

Mouse is a species that is commonly used in the study of asthma genetics. Most genome-scale association studies of asthma in mouse have been linkage studies that identified QTL for AHR [8], [9], [12], [13]. More recently, *in silico* QTL approaches have been employed. In a study that characterized baseline AHR in female mice from 36 strains and then used haplotype association mapping (HAM) to identify chromosomal regions associated with AHR, eight peaks on six chromosomes (i.e., 3, 5, 8, 12, 13, 14) with nominal association p-values were suggested as interesting for additional study [23]. The peak on chromosome 13 at 34.7 Mb was found to coincide with previous mouse AHR QTL results and was explored further to identify 29 candidate genes. Our mouse results do not support seven of the eight peaks found in the previous paper, but they do support the previous association on chromosome 13: our EMMA results had a peak of 25 SNPs with P-value 5.2e-04 in the range chr13∶36611438–36707895. Human associations for this region most strongly support the phenylalanyl-tRNA synthetase 2, mitochondrial (*FARS2*) gene as involved in asthma, with an EVE Latino American p-value of 6.4e-04 at rs9502304. However, the associations at this SNP were not significant among European Americans or African Americans, leading to a combined EVE meta-analysis p-value of 0.07 and exclusion as a top gene candidate in the current study. For the current study, some of the phenotype data reported by Leme *et al* [23] was utilized, but we extended this data by including AHR measures from male mice. Overall, we included data for 31 strains that had available AHR slope measures in mice of both genders and available genotype data. In addition to utilizing more phenotype data, we used a newer association method (i.e. EMMA) and more complete strain genotypic data than did the previous study. The primary advantage of EMMA vs. HAM is that EMMA takes the known genetic similarity between strains into account to control for population structure effects. The genome-wide genotype data for mouse strains used in the current study were for 281,300 SNPs, rather than 70,000 used in the previous report. Despite these improvements, our mouse association data still suffers from limitations. First, we are limited to a relatively small number of strains over which the range of AHR does not vary greatly. While the variability of AHR within strains seems to be significantly lower than that across strains, it may be the case that additional measures within strains would result in more precise phenotypes that have less within-strain variability and that increasing the number of strains phenotyped would provide a greater range of AHR variability across strains. Second, the phenotype studied, baseline AHR, may not be the best phenotype to characterize asthma susceptibility. For example, measures of AHR after mice are sensitized to ovalbumin may result in greater phenotype heterogeneity across strains and/or less heterogeneity within strains. Such limitations likely led the current mouse study to have insufficient statistical power to detect associations passing multiple comparisons correction. In addition to increasing the number of strains characterized and including additional phenotypes, future mouse genetic studies of asthma may be greatly improved with resources from the Collaborative Cross [38].

Translating mouse association results to the human genome is challenging at a genome-wide scale because of incomplete knowledge of the human/mouse genome map. We chose a gene-based analysis because the orthologous regions available in HomoloGene are the most accurately mapped ones, and also because such genes are most likely to be functionally relevant to phenotypes that are expressed in both mouse and human. It is possible that mouse genes excluded by the HomoloGene mapping are determinants of baseline AHR in mice. Thus, future studies with improved mouse/human genome maps may uncover additional regions of interest.

In summary, integration of genome-wide association results for a mouse strain survey of baseline AHR with human asthma and AHR GWAS results suggests that *KCNIP4* is a gene related to both asthma and AHR. Functional studies are required to validate the potential involvement of this gene in asthma and to understand how specific SNPs may modulate asthma susceptibility and/or AHR.

## Methods

### Mouse Airway Responsiveness Measures

A survey of AHR was conducted in males of 33 mouse strains, to augment a survey of AHR that was previously conducted in females of 36 mouse strains [23]. AHR was measured by gathering a baseline resistance reading for 6–10 mice per strain and gender, followed by a reading after administration of saline, and sequentially increasing concentrations of methacholine (1, 3, 10, 30 mg/ml) administered via nebulizer through a tracheostomy [23]. AHR was quantified as the slope of the resistance vs. log-transformed methacholine concentration.

### Mouse *in silico* Association Mapping

Of the 33 mouse strains with AHR phenotype data in males and the 36 mouse strains with AHR phenotype data in females, a subset of 31 strains overlapped and had genome-wide genotype data available. This subset of 31 mouse strains with AHR measures in both males and females and with available genotype data was used to conduct association analyses. The association of SNPs with AHR was measured using Efficient Mixed Model Association (EMMA) software [19], which uses a linear mixed model with a variance component using a kinship matrix that is based on the genetic similarity between strains to control for population structure effects. The genotype data for the mouse strains was part of the “Subspecific Origin and Haplotype Diversity in the Laboratory Mouse” project of the Jackson Laboratory available at http://cgd.jax.org/datasets/popgen/diversityarray/yang2011.shtml [39]. Autosomal chromosome association results obtained with male and female mice were utilized. Monomorphic and singleton SNPs were excluded, and the final dataset contained association results for 281,300 SNPs.

### Primary Human Asthma GWAS

The primary group of subjects consisted of participants of the EVE consortium, which comprises nine research teams in the USA with genome-wide association data for diverse asthma cohorts [26]. Briefly, 4,867 European Americans (1,486 cases, 1,539 controls, and 620 trios), 4,644 Latinos (606 cases, 792 controls, and 1,082 trios), and 3,447 African Americans (1,154 cases, 1,054 controls, and 413 trios) recruited through clinics and health systems in the U.S., Puerto Rico, Mexico, and Barbados were used in a meta-analysis of GWAS of asthma. The previously generated meta-analysis results for >2 million SNPs, based on genotyped or HapMap Phase 2 imputed data, for all subjects, as well as those limited to European American, African American, and Latino American groups were utilized. The test statistic to assess association of SNPs with asthma was based on a linear combination of normally distributed test statistics weighted by the square root of individual study sample sizes. Significance of this test statistic was assessed using standard normal approximations [26], [28].

### Replication Human Asthma GWAS

#### 1) Sepracor/LOCCS/LODO/Illumina

This cohort consisted of 531 non-Hispanic white cases with mild to severe asthma from three adult asthma populations: (1) a medication trial conducted by Sepracor, Inc., US [40], [41]; (2) the Leukotriene Modifier or Corticosteroid Salmeterol (LOCCS) study [42]; and (3) the Effectiveness of Low Dose Theophylline as an Add-on Treatment in Asthma (LODO) trial [43]. Cases were matched with 660 population controls obtained from Illumina’s IconDB resource (http://www.illumina.com/science/icontroldb.ilmn) using the genetic matching (GEM) algorithm [44]. Genome-wide genotyping of cases was performed on the Illumina 610 quad platform. Genome-wide genotyping of controls was performed on the Illumina HumanHap 550K v3 platform. Genotypes of SNPs of interest that were not captured by the genotype data were inferred using imputation with the Markov Chain Haplotyping software (MaCH) [45] based on HapMap Phase 2 Release 22 data [46]. The ratio of the empirically observed dosage variance to the expected (binomial) dosage variance for imputed SNPs utilized was greater than 0.3, indicating good quality of imputation. Dosage data was used to compute association statistics with PLINK [47]. The corresponding genetic inflation factor was 1.04, demonstrating minimal population stratification.

#### 2) GABRIEL

This European consortium-based GWAS of asthma consisted of 10,365 persons with physician-diagnosed asthma and 16,110 unaffected persons, all of whom were matched for ancestry [25]. Publicly available results for the random-effects pooled analysis that tested for association using the entire study population with asthma were used [Available at http://www.cng.fr/gabriel/results.html].

#### 3) Dutch asthma GWAS (DAG)

This cohort was comprised of 920 DAG subjects with doctor-diagnosed asthma and documented AHR and 985 controls [48], [49]. Genotyping was performed using the Hapmap 317K platform or Illumina 370 Duo Chip. Tests of association were performed via logistic regression using PLINK.

### Human AHR GWAS

#### 1) SHARP

A GWAS of AHR, quantified as the natural log of the dosage of methacholine causing a 20% drop in FEV_1_, was performed with 994 non-Hispanic white asthmatic subjects from the Childhood Asthma Management Program (CAMP) [50], and subsets of clinical trials within the Childhood Asthma Research and Education (CARE) network [51], and the Asthma Clinical Research Network (ACRN) [52] participating in the NHLBI SNP Health Association Resource Asthma Resource project (SHARP) [53]. Genome-wide SNP genotyping for SHARP subjects was performed by Affymetrix, Inc. (Santa Clara, CA) using the Affymetrix Genome-Wide Human SNP Array 6.0. Imputation of all SNPs available in HapMap Phase 2 Release 22 CEU data using MaCH [45] was performed. The primary GWAS was based on imputed SNPs with minor allele frequency (MAF) >0.05 and a ratio of empirically observed dosage variance to the expected (binomial) dosage variance greater than 0.3, indicating good quality of imputation. The association of SNPs with LnPC20 was measured with a linear regression model using dosage data as implemented in PLINK.

#### 2) Dutch asthma GWAS (DAG)

This cohort was comprised of 650 DAG subjects with doctor-diagnosed asthma and documented AHR [48], [49]. All subjects had smoking history and steroid use data available at the time of the AHR test. Participants with a history of AHR but in remission during the test were excluded. Remission was defined as not on steroids and without 20% or greater fall in FEV_1_ during the AHR test. The AHR test was conducted using histamine or methacholine as a stimulus. AHR was quantified as the difference between FEV_1_ at baseline and at the dose step at which a 20% or greater FEV_1_ drop was achieved, divided by the dose of stimulant used (slope). Because two protocols were used, one with a 30-second tidal breathing method and a second with a 2-minute tidal breathing phase, the AHR slopes measured with the 30-second tidal breathing method were divided by 4, in order to compensate for the 4 times greater duration of administration of stimulus. Slope values were log-transformed so that they would follow a normal distribution. Genotyping was performed using the Hapmap 317K platform or Illumina 370 Duo Chip. Tests of AHR association were performed via linear regression, with smoking and inhaled/oral steroid use as covariates using PLINK.

Each study was approved by its respective Institutional Review Board, which ensured that all procedures followed were in accordance with the ethical standards of the responsible committee on human experimentation. Informed consent was obtained for all study participants.

### Integration of Mouse and Human GWAS Results

Mouse gene coordinates were mapped to the human genome using the mouse comparative homology map data corresponding to Mouse Build 37.2, Human Build 37.2 and HomoloGene file from April 15, 2011 available at ftp://ftp.ncbi.nih.gov/pub/homology_maps/ [54]. After selection of orthologous genes, the list of genes was restricted based on having an EVE meta-analysis P-value <0.001 in one of the race/ethnic-specific analyses or in the combined GWAS. Plots of human association results near specific genes were created using LocusZoom with the hg18/HapMap Phase II CEU GenomeBuild/LD Population [55]. Combined P-values with replication populations were computed using Fisher’s combined probability method [56] where hypothesis tests in replication populations had one-sided alternatives, based on the direction of the association in EVE, so that SNPs with association tests in opposite directions would not produce inappropriately small P-values. Analyses were carried out using the R computing environment [57].
